# Comparison of Laparoscopic Percutaneous Extraperitoneal Internal Ring Closure by Two-Hook Hernia Needle and Open Repair for Pediatric Inguinal Hernia

**DOI:** 10.1089/lap.2022.0529

**Published:** 2023-08-07

**Authors:** Huaixiao Zhang, Yuan Feng, Jianguo Wang, Hongjun Zhao

**Affiliations:** Department of Pediatric Surgery, Xiangtan Central Hospital, Xiangtan, China.

**Keywords:** pediatric, inguinal hernia, laparoscopic, two-hook hernia needle, internal ring closure, open repair

## Abstract

**Purpose:**

In children, pediatric inguinal hernia (PIH) is a prevalent condition. PIH is currently more frequently managed by laparoscopic closure of the hernia sac. We improved this minimally invasive technique; that is, laparoscopic two-hook hernia needle percutaneous extraperitoneal internal ring closure. Safety and effectiveness were evaluated by comparing the differences between laparoscopic repair (LR) and open repair (OR) in terms of operation time, surgical complications, contralateral metachronous hernia incidence, and recurrence rate.

**Methods:**

A retrospective clinical data analysis was performed on pediatric patients who had hernia surgery utilizing the LR or OR method between June 2019 and June 2021. Medical records of all of the children were gathered, and clinical traits, information about the procedure, and follow-up were all analyzed.

**Results:**

A total of 370 patients' inguinal hernias were repaired. For 136 patients undergoing OR and 234 patients undergoing LR, all procedures were completed satisfactorily. There were 98 cases of bilateral hernias and 272 cases of unilateral hernias (180 on the right side and 92 on the left). In the LR group, 58 patients who had been initially diagnosed with unilateral hernias developed contralateral occult hernias intraoperatively. Inguinal hernia operations took an average of 13.82 (LR) and 32.07 (OR) minutes for unilateral cases, and 21.00 (LR) and 54.85 (OR) minutes for bilateral cases. For LR and OR, the average follow-up time was 22.41 months and 23.10 months, respectively. The perioperative complications included peritoneal rupture in 3 patients, scrotal edema or hematoma in 5, hydrocele in 3, and groin pain in 6. In the LR group, 1 patient experienced the postoperative recurrence, whereas 8 individuals in the OR group did.

**Conclusions:**

Our initial research showed that laparoscopic two-hook hernia needle percutaneous extraperitoneal internal ring closure inguinal hernia repair is a safe and effective procedure. The LR method has the benefits of concealing the incision, a quicker procedure, having a lower risk of complications, and finding contralateral patent processus vaginalis. Therefore, promoting and using this surgical technique in clinical practice are merited. Clinical Trial Registration number: Medical Association of Xiangtan (2022-xtyx-28)

## Introduction

One of the most frequent surgical diseases in children is pediatric inguinal hernia (PIH), with incidence rates ranging from 0.8% to 4.4%.^1^ For the majority of pediatric surgeons, the gold-standard treatment for hernias is still traditional open surgery. Open repair (OR) has been used for more than 100 years, and recently published research shows that the recurrence rate is low, ranging from 0% to 6%.^2,3^ However, some complications might occur, including surgical site infection, iatrogenic cryptorchidism, hematoma, hydrocele, spermatic cord injury, vas deferens injury, and testicular atrophy.^4^

Laparoscopic percutaneous extraperitoneal internal ring closures have been documented for more than 10 years.^5^ Internal ring closure by the laparoscopic percutaneous extraperitoneal method has become increasingly common recently.^6^ With the decreasing use of working ports and endoscopic instruments and the increasing use of extracorporeal knotting, techniques continue to evolve.^7^ Nowadays, LR has been increasingly used to manage PIH. Compared with OR, laparoscopic surgery has significant benefits, including the finding of contralateral abnormalities, avoiding excessive spermatic cord dissection, quick recovery, improving cosmesis, and reducing complications.^8^

Nevertheless, the previous single-port laparoscopic percutaneous extraperitoneal internal ring closure technique was better suited for seasoned surgeons. For beginners, coordination issues with their eyes and hands as well as difficulties with depth perception were present. To reduce the difficulty observed in surgical technique, we improved the use of surgical instruments such that the two-hook hernia needle apparatus can be completely operated by one hand during the operation. When we improved the technique and used the two-hook hernia needle apparatus, laparoscopic repair (LR) became simpler and faster, which shortened the learning curve of this advanced technique. The purpose of this study was to evaluate the safety and effectiveness of LR and OR by comparing their differences in operation time, surgical complications, contralateral metachronous hernia (CMH) incidence, and recurrence rate.

## Materials and Methods

### Clinical data

This retrospective study included 387 pediatric patients who had hernia repairs utilizing the LR or OR method between June 2019 and June 2021. Parents or other adult guardians of the children signed informed consent forms, and our Institutional Review Board authorized this study. Both the LR and OR surgical methods were fully explained so that the parents of the children could choose the one they felt most comfortable with. The diagnosis was confirmed in all cases after a physical examination and preoperative ultrasound scan.

Patients between the ages of 8 months and 14 years who underwent inguinal hernia repair using the LR technique or the OR technique and were monitored for >6 months after surgery met the inclusion criteria. Patients <8 months or >14 years old, lost to follow-up, or who had been observed for <6 months, as well as those with incarcerated hernias or other diseases needing concurrent surgical treatment, were excluded from the study. The data collected included medical history, demographic information, clinical characteristics, surgical treatment outcomes, postoperative complications, and recurrence.

### Surgical instrument

**FIG. 1.**
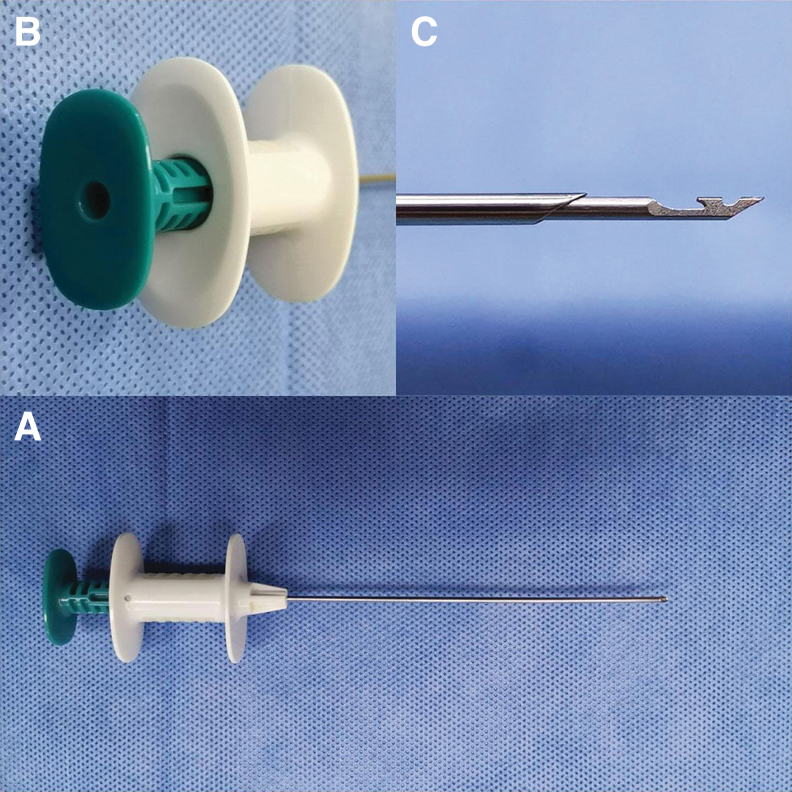
The two-hook hernia needle instrument. **(A)** The appearance of the two-hook hernia needle apparatus. **(B)** The apparatus contains a hole in the tail end that may be used to attach a syringe. **(C)** Two slots in the core of the two-hook hernia needle.

The two-hook hernia needle apparatus was produced by Xiamen Surgaid Medical Equipment Co., Ltd. of China (Fig. 1). To assist blunt dissection of the preperitoneal area, the double trocar used for the puncture had a blunt tip and a curved, spatulate front end. In addition, it contains a hole in the tail end that may be used to attach a syringe. These features allowed for intraoperative injection of normal saline to divide the preperitoneal area, and reduce damage to the testicular ducts and spermatic veins.

### Surgical method

#### LR method

**FIG. 2.**
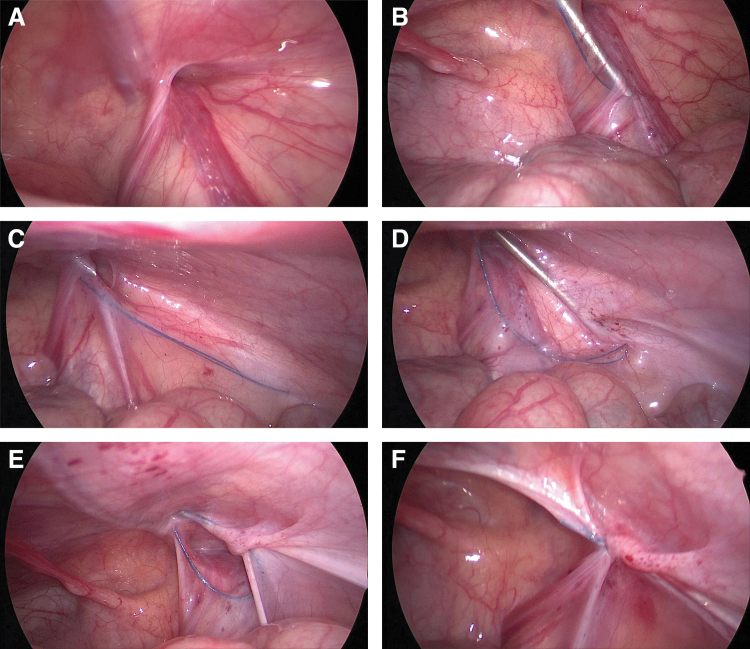
Operation steps for laparoscopic percutaneous extraperitoneal internal ring closure by a two-hook hernia needle. **(A)** Explore the internal ring opening of hernia and the condition of the contralateral inner ring opening. **(B)** The hernia needle was passed through the peritoneal cavity lateral to the vas deferens or the round ligament of uterus. **(C)** After the hernia needle passed through the 6 o'clock position of the internal inguinal ring, the nonabsorbable suture is retained in the extraperitoneal space. **(D)** The nonabsorbable thread is hooked by the hernia needle and removed from the extraperitoneal space. **(E)** The suture encircles the hernia sac neck in the extraperitoneal space. **(F)** The internal ring opening was ligated and checked laparoscopically.

The patients were positioned in a supine position with their head low and their feet high ∼15° to 20°, after general anesthesia. A 5-mm curved incision was made around the umbilicus after the surgical instruments had been prepared. A 5-mm trocar was introduced after establishing the pneumoperitoneum. In accordance with the child's weight and age, the intra-abdominal pressure was regulated between 6 and 12 mmHg. A 5-mm, 30° laparoscope was inserted, the entire abdominal cavity was examined, the condition of the internal ring was observed (Fig. 2A), and the contralateral inguinal internal ring opening was explored.

On the side of the hernia, a 1 mm incision was made above the internal ring. In the two-hook hernia needle, the 3-0 Prolene (polypropylene seam nonabsorbable suture) string hook was positioned in the groove. The hernia needle was inserted through a small incision in the groin into the preperitoneal space under the close monitoring of a laparoscope. The hernia needle was pushed forward extraperitoneally along the inside of the internal inguinal ring until it passed through the vas deferens and spermatic vessels (Fig. 2B).

The “hydrodissection” approach can be used to separate the extraperitoneal space when it was difficult to separate the spermatic vessels and vas deferens from the peritoneum, minimizing damage to the vessels and vas.^9^ When the hernia needle passed through the 6 o'clock position of the inner inguinal ring, it continued to separate and moved forward about 2–3 cm, and the nonabsorbable suture was retained in the extraperitoneal space (Fig. 2C).

The hernia needle was slowly withdrawn along the original path to the extraperitoneal space to the 12 o'clock position of the internal inguinal ring, but it cannot retreat to the muscular layer. After that, the orientation of the hernia needle was changed and passed along the lateral side of the inner ring opening to the extraperitoneal area's 6 o'clock position, and the hernia needle was used to clamp the nonabsorbable thread that was preset here (Fig. 2D). And the head end of the suture was drawn out of the groin's tiny incision. The suture encircles the hernia sac neck in the extraperitoneal space (Fig. 2E).

After the air being forced out of the hernia sac, a knot was knotted *in vitro*, and the knot was inserted into the extraperitoneal space beneath the muscular layer. Finally, the ligation of the inner inguinal ring was inspected by laparoscopy (Fig. 2F). The same procedure was performed on the contralateral side if there was CMH. To conclude the procedure, the pneumoperitoneum was relieved, the trocar was removed, the 5-0 absorbable sutures were used to stitch the umbilical incision, the tiny incision in the groin was adhered with medical adhesion agent. Extreme caution was exercised throughout the operation to prevent harm to the inferior epigastric, femoral, and external iliac vessels. In addition, it is crucial to ensure the extraperitoneal hernia sac is completely ligated, and the hernia needle cannot penetrate the peritoneum into the abdominal cavity.

#### OR method

Under general anesthesia, the patient was lying in a supine position. In OR, a 1 cm transverse incision was made along the dermatoglyph in the symptomatic inguinal region, and a high ligation was only performed on the problematic side. The hernia sac was found in the anteromedial region of the testicular cord, which was dissected and separated, and then was ligated at a high level at the opening of the internal ring. When cutting, separating, and ligating the hernia sac, special care should be taken to avoid damaging the testicular arteries and vas deferens.

### Follow-up

Intraoperative (conversion, peritoneum rupture) and postoperative complications (surgical site infection, scrotal edema, hydrocele, groin pain, ascending testis, testicular atrophy, recurrence) were documented in the study scheme. Almost every case (98%) was a day surgery case. The first visit was 1 week after operation at our outpatient clinic. Follow-up was completed every 3 months in the later period.

### Statistical analysis

Data analysis was performed using SPSS 26.0 software (IBM Corp, Armonk, NY). To describe continuous variables, values are expressed as mean-SD. For categorical variables, absolute and relative frequencies were used. The independent samples *t* test was used to compare the distribution of quantitative variables between groups. Chi-square or Fisher's exact test was used to compare the significant differences of the categorical variables between groups.

## Results

### Patients

Seventeen patients who met at least one of the exclusion criteria were not included in the research. In the end, 370 patients were involved in the research. All treatments were successfully carried out for the 234 patients who had LR and the 136 patients who received OR in our center. During the period of our study, the youngest patient who underwent surgery was 8 months old and the oldest was 14 years old; the mean age at operation was 37.21 months, including 67 females and 303 males.

In the LR group, 58 patients who had been originally diagnosed with unilateral hernias developed contralateral occult hernias intraoperatively. A bilateral hernia occurred in 98 cases, and a unilateral hernia occurred in 272 instances (180 on the right and 92 on the left). The clinical statistical data characteristics of the two groups are indicated in Table 1. There were no significant differences between the two groups in basic characteristics such as age, sex, and laterality before operation.

**Table 1.** The Laparoscopic Repair and Open Repair Groups' Clinical Statistics Data Characteristics Before Surgery

Characteristics	LR	OR	*t* (χ^2^) value	*P *value
	*n* = 234	*n* = 136		
Age (months)	36.65 ± 22.17	37.76 ± 24.12	*t* = 0.451	.218
Gender (%)				
Male	188 (80.3)	115 (84.6)	*χ*^2^ = 1.031	.31
Female	46 (19.7)	21 (15.4)		
Laterality (preoperative) (%)				
Right	136 (58.1)	78 (57.4)	*χ*^2^ = 0.131	.949
Left	72 (30.8)	44 (32.3)		
Bilateral	26 (11.1)	14 (10.3)		

LR, laparoscopic repair; OR, open repair.

### Operation

There were 150 patients with unilateral inguinal hernia surgery and 84 patients with bilateral inguinal hernia surgery in the LR group. In this series, contralateral patent processus vaginalis (CPPV) was discovered intraoperatively in 58 of 208 cases with unilateral inguinal hernias who had preoperative diagnoses, with an incidence rate of 27.9%. Thus, a total of 318 (150 unilateral and 84 bilateral) repairs were performed in the LR group. All LR patients had successful procedures without the necessity for open conversion. In the OR group, 122 people had surgery for a single inguinal hernia, and 14 people had surgery for a double inguinal hernia. Interestingly, Table 1 shows that there was no significant difference in surgical lateral position between the two groups before surgery (*P* > .949). However, there was a significant difference in surgical lateral position between the two groups after operation (*P* < .001) (Table 2).

**Table 2.** Comparison of the Surgical and Treatment Outcomes Between the Two Groups

Characteristics	LR	OR	*t* (χ^2^) value	*P *value
Operation time (min)				
Unilateral	13.82 ± 1.89	32.07 ± 4.27	*t* = 47.18	<.001
Bilateral	21.00 ± 2.11	54.85 ± 3.18	*t* = 42.21	<.001
Laterality (postoperative) (%)				
Right	102 (43.6)	78 (57.4)	*χ*^2^ = 29.48	<.001
Left	48 (20.5)	44 (32.3)		
Bilateral	84 (35.9)	14 (10.3)		
Hospital stay (day)	1 (IQR1,1)	1 (IQR1,1)	—	>.999
Conversion (%)	0	0	—	—
Follow-up period (months)	22.41 ± 7.37	23.10 ± 7.99	*t* = 0.836	.404

The average operation time for LR group was 13.82 ± 1.89 minutes for unilateral hernia repair and 21.00 ± 2.11 minutes for bilateral hernia repair. In OR group, the average surgery time for unilateral was 32.07 ± 4.27 minutes and that for bilateral was 54.85 ± 3.18 minutes. For both unilateral and bilateral hernia repairs, operation time was significantly reduced in the LR group (*P* < .001). Since most (98%) of the patients underwent daytime surgery, all patients were discharged 1 day after surgery, so there was no significant difference in postoperative hospital stay between the two groups (*P* > .999). The mean follow-up period was 22.41 ± 7.37 months for LR and 23.10 ± 7.99 months for OR. The follow-up time showed no significant difference between the two groups (Table 2).

### Complications

In this study, there were 26 cases with complications (Table 3). Three patients (2.2%) were found to have peritoneal rupture after surgery in OR group, and the incidence of peritoneal rupture was significantly different between the two groups (*P* = .049). In comparison with the LR group (0), the OR (3.7%) group had a significantly higher frequency of scrotal hematoma and seroma, and the difference was statistically significant (*P* = .006). They were resolved with physiotherapy and observation.

**Table 3.** Comparison of the Laparoscopic Repair and Open Repair Groups' Postoperative Complications and Recurrence

Characteristics	LR	OR	*t* (χ^2^) value	*P *value
Complications (%)				
Peritoneal rupture	0	3 (2.2)	*χ*^2^ = 5.204	.049
Surgical site infection	0	0	—	—
Scrotal edema/hematoma	0	5 (3.7)	*χ*^2^ = 8.721	.006
Hydrocele	2 (0.85)	1 (0.74)	*χ*^2^ = 0.015	.902
Groin pain	0	6 (4.4)	*χ*^2^ = 10.494	.002
Ascending testis	0	0	—	—
Testicular atrophy	0	0	—	—
Recurrence (%)				
Ipsilateral recurrent hernia	1 (0.43)	1 (0.74)	*χ*^2^ = 0.152	1
Contralateral metachronous hernia	0	7 (5.1)	*χ*^2^ = 12.276	.001

Hydrocele developed in 3 patients, of which 1 (0.74%) in OR and 2 (0.85%) in LR, and there were no significant differences between the two groups (*P* = .902). Two cases of hydrocele resolved spontaneously within 2 months, while the other required a testicular sheath inversion procedure. There were six cases of postoperative groin pain in the OR group and none in the LR group; the incidence of postoperative groin pain in the OR group (4.4%) was higher than that in the LR group (0), and the difference was statistically significant between the two groups (*P* = .002).

In the OR group and the LR group, ipsilateral hernia recurrences happened once each throughout the follow-up period. Between the two groups, there was no significant difference in the frequency of ipsilateral hernia recurrence (*P* = 1.00). Statistical results showed that CMH occurred in seven cases in the OR group and in 0 cases in the LR group (Table 3); the incidence of CMH in the OR group was significantly higher than that in the LR group (*P* = .001). No surgical site infection, ascending testis, and testicular atrophy developed in either group.

## Discussion

For children with inguinal hernias, surgery is currently the most effective treatment. Open surgery is the gold standard for treating PIHs and one of the most common treatments for children.^10^ However, laparoscopic surgical methods have recently become the first choice for PIHs treatment.^11^ Not only can it lessen the apparent skin incision of conventional surgery and enhance the esthetic results,^12^ but it also has more advantages than open hernia repair in terms of operative time, postoperative complications, and recurrent hernia rate. In this study, we found that more and more parents choose laparoscopic hernia repair, and this phenomenon is becoming increasingly obvious.

There are various reports on the length of time for laparoscopic hernia repair. According to some research, laparoscopic hernia repairs take longer to complete than open ones,^13^ while other studies found no difference between the two,^14^ and still other studies found the opposite.^15^ In our study, laparoscopic surgery took 13.82 minutes for unilateral and 21.00 minutes for bilateral inguinal hernias.

According to our study, the average time for an open inguinal hernia procedure was 32.07 minutes for unilateral and 54.85 minutes for bilateral. Compared with the LR group, our findings demonstrated that the OR group's surgery time was significantly longer (*P* < .001), both unilateral and bilateral. Similar findings were reported in other literature.^16,17^ These findings are similar to those of multiple other studies,^18^ and support the notion that LR is quicker to operate than OR in PIH repair.

The operation time is not only related to the surgeon's experience but also to the continuous development of various minimally invasive surgical instruments. Several studies have suggested that when a surgeon becomes more skilled with laparoscopy, there are fewer complications, fewer recurrences, and shorter operating times.^19^ The two-hook hernia needle substantially decreases the surgical time because it does not require skilled intracorporeal suturing and knot tying, and the inguinal canal does not require dissection.

Compared with OR, the significant advantage of laparoscopic surgery is that CMH and CPPV can be detected simultaneously.^20^ However, there is still controversy regarding contralateral inguinal exploration in children, and whether CPPV should be ligated at the same time.^21^ Some studies suggest that patients with CPPV have a low probability of progressing to CMH, and that there is no need for therapy.^22,23^ These scholars believe that contralateral exploration has the risk of damaging the vas deferens, spermatic vessels, and testis, as well as the disadvantages of increasing the operation time and anesthesia time.^24^

In fact, surgeons are increasingly using laparoscopic exploration of the contralateral groin to prevent CMH that may occur at any time.^25^ Contralateral groin exploration and simultaneous ligation will reduce recurrence risk, parental concerns, additional surgery, and anesthesia rather than raising risk or resulting in additional harm.^26^ In the LR group, when LR for unilateral inguinal hernias was performed, CPPV was discovered in 27.9% of patients, which was ligated concurrently. Throughout the follow-up period, we found that the frequency of CMH in LR group (0) was significantly lower than that in OR group (5.1%).

At present, there are many different reports about the recurrence rate of laparoscopic hernia repair and OR. Some studies have demonstrated that the recurrence risk for laparoscopic hernia repair ranges from 0% to 5%.^27^ However, other studies have shown that the recurrence risk in PIH surgery ranges from 0% to 6% in OR and 0% to 15.5% in LR.^28^ Yang et al^29^ reported in their meta-analysis that two studies found laparoscopic hernia repair had a higher recurrence rate than OR.^30,31^

Laparoscopic procedures have been proven in three studies to lower the recurrence rate after surgery,^32–34^ while no differences between the two were found in two additional studies.^35,36^ In our study, we observed an interesting result that the recurrence rate of ipsilateral recurrent hernia is different from that of CMH. In our research, ipsilateral recurrent hernia occurred in 1 of 234 cases in the LR group and 1 of 136 cases in the OR group, while we observed a CMH rate of 0 in LR and 5.1% in OR. Between the two groups, there was no discernible difference in the incidence of ipsilateral recurrent hernia (*P* = 1.00), but this study demonstrated that the CMH rate after LR was lower than that after OR, and the difference was significant (*P* = .001).

Recurrence may occur as a result of insufficient internal ring ligation, inguinal canal injury from surgical trauma, a thin and easily burst sac, postoperative wound infection, and hematoma.^33^ In the LR technique, recurrence may occur if a sizable section of the patent processus vaginalis (PPV) is skipped and an absorbable suture is applied.^37^ Since the hernial sac wall is rather thin and is likely to avulse during dissection, open herniorrhaphy for children is sometimes challenging. In contrast, the LR technique does not require dissecting the inguinal canal to approach the internal ring. The shortened operating time and the low frequency of recurrence in LR may be attributed to this straightforward method.

In the present series, two patients in the LR group had postoperative hydrocele after 1 month. For the OR group in this study, there was only one postoperative hydrocele (*P* = .902). Because the PPV is only ligated at the deep ring and the distal part of the hernia sac was left intact in LR, hydrocele was a potential complication; consequently, a residual hydrocele might be more noticeable after surgery.^7^ However in OR, the PPV is often separated and any leftover hydrocele is drained. Similar results were observed by Saha et al,^38^ who found that there was a statistically insignificantly higher incidence of hydrocele after LR than after OR, but none of these cases underwent surgery.

In this study, we noted that the OR group had a significantly higher incidence of scrotal edema or hematoma than the LR group (*P* = .006). Nazem et al^39^ similarly observed that OR increased the rate of scrotal edema and hematoma compared with LR. The main reason for this complication was that more dissection was performed along the inguinal canal in the OR group, while the LR group did not need to dissect the inguinal canal. In addition, excessive dissection may injure the spermatic arteries and testicular duct, as well as cause the hernial sac wall to avulse. Some studies point out that the majority of the scrotal edema can be naturally absorbed,^40^ and we only need to do some simple treatments such as physical therapy and observation.

Other infrequent issues with LR included wound infection, testicular atrophy, and ascending testis.^8^ It was satisfactory that the LR and OR groups hardly observed them, perhaps due to the increasing precision of operations and strict implementation of aseptic technology. LR has many benefits, including less postoperative pain, a quick return to normal activities, and outstanding cosmetic results, in addition to taking less time to perform and having fewer surgical complications.^41^

In our study, there were no complications related to groin pain in the LR group; however, six cases (4.4%) developed groin pain in the OR group, and all of these patients had scrotal swelling. Our experience is that it is linked with scrotal hematoma, because the purpose of open surgery is to perform a high ligation of the hernia sac after finding it by dissecting the inguinal region. Therefore, excessive dissection and disassociation are probable to result in avulsion of the hernial sac wall and vascular injury to the inguinal area. None of the LR patients in our study were converted to OR, which is similar to the findings in the majority of the literature.^42,43^

There are also certain limitations observed in this study. For example, this was retrospective research, so there was some selection and recall bias between the LR and OR groups, which probably affected the results of our analyses. The cases in this study were from a single center, and the sample size was small, so there could be some variance between the statistical findings and the multicenter research. In addition, the relatively brief follow-up period likely understates the frequency of some surgical complications, such as testicular atrophy and ascending testis. Although our study showed that LR had superior results in the short- to mid-term period compared with OR, the long-term results of LR need further observation. Therefore, to further examine the potential causes of all complications, we will keep collecting follow-up data in our future studies.

## Conclusion

In conclusion, we can observe that LR and OR were reliable and efficient ways to treat PIH. According to the outcomes of our clinical cases, laparoscopic two-hook hernia needle percutaneous extraperitoneal internal ring closure is a secure and doable treatment. Compared with OR, the LR method has the benefits of incision concealment, shorter surgery time, CPPV detection, and a lower incidence of complications. This procedure could be considered minimally invasive surgery in a genuine sense because it is secure, easy, and less invasive. So, promoting and using this surgical technique in clinical practice are worthwhile.

## Ethics Approval

All procedures performed in studies involving human participants were in accordance with the ethical standards of the institutional and/or national research committee, and with the 1964 Helsinki declaration and its later amendments or comparable ethical standards. Parents or other adult guardians of the children signed informed consent forms, and our Institutional Review Board (Medical Association of Xiangtan, 2022-xtyx-28) authorized this study.

## Human and Animal Rights

This article is a retrospective study on human, and does not involve animal. We followed compliance with ethical standards during the study.

## Consent for Publication

Parents or other adult guardians of the children have signed the informed consent form before surgery. In this study, there are no other unidentified personal or clinical details along with other unidentified images to be published.

## Authors' Contribution

HXZ provided conception and design of study; YF and HJZ performed acquisition of data; HXZ, YF, and JGW contributed to data analysis and interpretation; HXZ and JGW assisted with drafting of the article and critical revision; HXZ and HJZ provided approval of final version of the article; the article's submission was reviewed and approved by all authors.

## References

[1] Zhu LL, Xu WJ, Liu JB, et al. Comparison of laparoscopic hernia repair and open herniotomy in children: A retrospective cohort study. Hernia 2017;21(3):417–423; doi: 10.1007/s10029-017-1607-x 28424930

[2] Esposito C, Giurin I, Alicchio F, et al. Unilateral inguinal hernia: Laparoscopic or inguinal approach. Decision making strategy: A prospective study. Eur J Pediatr 2012;171:989–991. 22350286 10.1007/s00431-012-1698-4

[3] Nah SA, Giacomello L, Eaton S, et al. Surgical repair of incarcerated inguinal hernia in children: Laparoscopic or open? Eur J Pediatr Surg 2011;21:8–11. 20938898 10.1055/s-0030-1262793

[4] Esposito C, Montinaro L, Alicchio F, et al. Technical standardization of laparoscopic herniorraphy in pediatric patients. World J Surg 2009;33:1846–1850. 19597875 10.1007/s00268-009-0121-4

[5] Van Batavia JP, Tong C, Chu DI, et al. Laparoscopic inguinal hernia repair by modifed peritoneal leafet closure: Description and initial results in children. J Pediatr Urol 2018;14(3):2721–2716; doi: 10.1016/j.jpurol.2018.02.015 PMC6084465 29958645

[6] Zhan Y, Chao M, Zhang X, et al. Does the laparoscopic treatment of paediatric hydroceles represent a better alternative to the traditional open repair technique? A retrospective study of 1332 surgeries performed at two centres in China. Hernia 2018;22:661–669; doi: 10.1007/s10029-017-1715-7 29243214 PMC6061066

[7] Saranga BR, Arora M, Baskaran V. Minimal access surgery of pediatric inguinal hernias: A review. Surg Endosc 2008;22:1751–1762. 18398652 10.1007/s00464-008-9846-7

[8] Esposito C, Escolino M, Turra` F, et al. Current concepts in the management of inguinal hernia and hydrocele in pediatric patients in laparoscopic era. Semin Pediatr Surg 2016;25:232–240. 27521714 10.1053/j.sempedsurg.2016.05.006

[9] Tatekawa Y. Laparoscopic extracorporeal ligation of hernia defects using an epidural needle and preperitoneal hydrodissection. J Endourol 2012;26:474–477. 22168769 10.1089/end.2011.0498 PMC3331728

[10] Forte A, D'Urso A, Palumbo P, et al. Inguinal hernioplasty: The gold standard of hernia repair. Hernia 2003;7:35–38; doi: 10.1007/s10029-002-0095-8 12612796

[11] Liem MS, van Duyn EB, van der Graaf Y, et al. Recurrences after conventional anterior and laparoscopic inguinal hernia repair: A randomized comparison. Ann Surg 2003;237:136–141; doi: 10.1097/00000658-200301000-00019 12496541 PMC1513978

[12] Takehara H, Yakabe S, Kameoka K. Laparoscopic percutaneous extraperitoneal closure for inguinal hernia in children: Clinical outcome of 972 repairs done in 3 pediatric surgical institutions. J Pediatr Surg 2006;41(12):1999–2003; doi: 10.1016/j.jpedsurg.2006.08.032. 17161191

[13] Zhu H, Li J, Peng X, et al. Laparoscopic percutaneous extraperitoneal closure of the internal ring in pediatric recurrent inguinal hernia. J Laparoendosc Adv Surg Tech A 2019;29(10):1297–301; doi: 10.1089/lap.2019.0119 31393202

[14] Igwe AO, Talabi AO, Adisa AO, et al. Comparative Study of Laparoscopic and Open Inguinal Herniotomy in Children in Ile Ife, Nigeria: A Prospective Randomized Trial. J Laparoendosc Adv Surg Tech A 2019;29(12):1609–1615; doi: 10.1089/lap.2019.0354. 31647350

[15] Shalaby R, Ibrahem R, Shahin M, et al. Laparoscopic hernia repair versus open herniotomy in children: A controlled randomized study. Minim Invasive Surg 2012;2012:484135. 23326656 10.1155/2012/484135 PMC3543810

[16] Li S, Li M, Wong KK, et al. Laparoscopically assisted simple suturing obliteration (LASSO) of the internal ring using an epidural needle: A handy single-port laparoscopic herniorrhaphy in children. J Pediatr Surg 2014;49:1818–1820; doi: 10.1016/j.jpedsurg.2014.09.027 25487491

[17] Abd-Alrazek M, Alsherbiny H, Mahfouz M, et al. Laparoscopic pediatric inguinal hernia repair: A controlled randomized study. J Pediatr Surg 2017;52:1539–1544; doi: 10.1016/j.jpedsurg.2017.07.003 28751002

[18] Ho IG, Ihn K, Koo EJ, et al. Laparoscopic repair of inguinal hernia in infants: Comparison with open hernia repair. J Pediatr Surg 2018;53:2008–2012. 29477445 10.1016/j.jpedsurg.2018.01.022

[19] Bracale U, Merola G, Sciuto A, et al. Achieving the learning curve in laparoscopic inguinal hernia repair by Tapp: A quality improvement study. J Invest Surg 2019;32:738–745; doi: 10.1080/08941939.2018.1468944 29902096

[20] Yonggang H, Changfu Q, Ping W, et al. Single-port laparoscopic percutaneous extraperitoneal closure of inguinal hernia using “two-hooked” core needle apparatus in children. Hernia 2019;23(6):1267–1273; doi: 10.1007/s10029-019-01933-9 30993474

[21] Hoshino M, Sugito K, Kawashima H, et al. Prediction of contralateral inguinal hernias in children: A prospective study of 357 unilateral inguinal hernias. Hernia 2014;18:333–337. 23644774 10.1007/s10029-013-1099-2 PMC4037557

[22] Miltenburg DM, Nuchtern JG, Jaksic T, et al. Laparoscopic evaluation of the pediatric inguinal hernia: A metaanalysis. J Pediatr Surg 1998;33:874–879. 9660219 10.1016/s0022-3468(98)90664-9

[23] Kokorowski PJ, Wang HH, Routh JC, et al. Evaluation of the contralateral inguinal ring in clinically unilateral inguinal hernia: A systematic review and meta-analysis. Hernia 2014;18(3):311–324; doi: 10.1007/s10029-013-1146-z 23963735 PMC3931747

[24] Nataraja RM, Mahomed AA. Systematic review for paediatric meta chronous contralateral inguinal hernia: A decreasing concern. Pediatr Surg Int 2011;27:953–961. 21604078 10.1007/s00383-011-2919-z

[25] Li S, Liu X, Wong KKY, et al. Single-port laparoscopic herniorrhaphy using a two-hooked cannula device with hydrodissection. J Pediatr Surg 2018;53(12):2507–2510; doi: 10.1016/j.jpedsurg.2018.08.009 30227994

[26] Holcomb GW 3rd, Miller KA, Chaignaud BE, et al. The parental perspective regarding the contralateral inguinal region in a child with a known unilateral inguinal hernia. J Pediatr Surg 2004;39:480–482. 15017573 10.1016/j.jpedsurg.2003.11.018

[27] Shalaby R, Elsayaad I, Alsamahy O, et al. One trocar needlescopic assisted inguinal hernia repair in children: A novel technique. J Pediatr Surg 2017;53:192–198; doi: 10.1016/j.jpedsurg.2017.08.020 28947323

[28] Kang CH, Kim YJ, Kim KT. Initial experience with percutaneous internal ring suturing for indirect inguinal hernia in pediatric patients. J Minim Invasive Surg 2020;23:67–73. 35600061 10.7602/jmis.2020.23.2.67 PMC8985616

[29] Yang C, Zhang H, Pu J, et al. Laparoscopic vs open herniorrhaphy in the management of pediatric inguinal hernia: A systemic review and meta-analysis. J Pediatr Surg 2011;46 (9):1824–1834. 21929997 10.1016/j.jpedsurg.2011.04.001

[30] Koivusalo AI, Korpela R, Wirtavuori K, et al. A single-blinded, randomized comparison of laparoscopic versus open hernia repair in children. Pediatrics 2009;123 (1):332–337. 19117900 10.1542/peds.2007-3752

[31] Hassan ME, Mustafawi AR. Laparoscopic flip-flap technique versus conventional inguinal hernia repair in children. JSLS 2007;11 (1):90–93. 17651564 PMC3015781

[32] Tsai YC, Wu CC, Yang SS. Open versus minilaparoscopic herniorrhaphy for children: A prospective comparative trial with midterm follow-up evaluation. Surg Endosc 2010;24 (1):21–24. 19690916 10.1007/s00464-009-0645-6

[33] Endo M, Watanabe T, Nakano M, et al. Laparoscopic completely extraperitoneal repair of inguinal hernia in children: A single-institute experience with 1,257 repairs compared with cut-down herniorrhaphy. Surg Endosc 2009;23 (8):1706–1712. 19343444 10.1007/s00464-008-0300-7 PMC2710496

[34] Niyogi A, Tahim AS, Sherwood WJ, et al. A comparative study examining open inguinal herniotomy with and without hernioscopy to laparoscopic inguinal hernia repair in a pediatric population. Pediatr Surg Int 2010;26 (4):387–392. 20143077 10.1007/s00383-010-2549-x

[35] Chan KL, Hui WC, Tam PK. Prospective randomized single-center, single-blind comparison of laparoscopic vs open repair of pediatric inguinal hernia. Surg Endosc 2005;19 (7):927–932. 15920685 10.1007/s00464-004-8224-3

[36] Saranga BR, Arora M, Baskaran V. Pediatric inguinal hernia: Laparoscopic versus open surgery. JSLS 2008;12 (3):277–281. 18765052 PMC3015882

[37] Shalaby R, Essa AG, Yehya AA, et al. Laparoscopic hernia repair in infancy and childhood: Evaluation of two different techniques. Ann Pediatr Surg 2010;6:6–13. 10.1016/j.jpedsurg.2010.07.004 21034946

[38] Saha N, Biswas I, Rahman M, et al. Surgical outcome of laparoscopic and open surgery of pediatric inguinal hernia. Mymensingh Med J 2013;22:232–236. 23715341

[39] Nazem M, Dastgerdi MMH, Sirousfard M. Outcomes of pediatric inguinal hernia repair with or without opening the external oblique muscle fascia. J Res Med Sci 2015;20:1172–1176. 26958052 10.4103/1735-1995.172985 PMC4766824

[40] Ibrahim M, Getso K, Mohammad M, et al. Herniotomy in resource-scarce environment: Comparison of incisions and techniques. Afr J Paediatr Surg 2015;12:45. 25659550 10.4103/0189-6725.150980 PMC4955508

[41] Korkmaz M, Güvenç BH. Comparison of single-port percutaneous extraperitoneal repair and three-port mini-laparoscopic repair for pediatric inguinal hernia. J Laparoendosc Adv Surg Tech A 2018;28:337–342. 29048979 10.1089/lap.2016.0223

[42] McClain L, Streck C, Lesher A, et al. Laparoscopic needle-assisted inguinal hernia repair in 495 children. Surg Endosc 2015;29:781–786. 25106720 10.1007/s00464-014-3739-8

[43] Hamad MA, Osman MA, Abdelhamed M. Laparoscopicassisted percutaneous internal ring ligation in children. Ann Pediatr Surg 2011;7:66–69.

